# Cervical, Anal and Oral HPV in an Adolescent Inner-City Health Clinic Providing Free Vaccinations

**DOI:** 10.1371/journal.pone.0037419

**Published:** 2012-05-18

**Authors:** Nicolas F. Schlecht, Robert D. Burk, Anne Nucci-Sack, Viswanathan Shankar, Ken Peake, Elizabeth Lorde-Rollins, Richard Porter, Lourdes Oriana Linares, Mary Rojas, Howard D. Strickler, Angela Diaz

**Affiliations:** 1 Department of Epidemiology and Population Health, Albert Einstein College of Medicine, Bronx, New York, United States of America; 2 Department of Pediatrics, Microbiology and Immunology, Albert Einstein College of Medicine, Bronx, New York, United States of America; 3 Department of Pediatrics, Mount Sinai Adolescent Health Center, Mount Sinai School of Medicine, Manhattan, New York, United States of America; 4 Department of Pediatrics, Infants and Children's Hospital, Maimonides Medical Center, Brooklyn, New York, United States of America; 5 Department of Obstetrics and Gynecology and Women's Health, Albert Einstein College of Medicine, Bronx, New York, United States of America; IPO, Inst Port Oncology, Portugal

## Abstract

**Objectives:**

Published human papillomavirus (HPV) vaccine trials indicate efficacy is strongest for those naive to the vaccine-types. However, few high-risk young women have been followed and cervical HPV has been the predominant outcome measure.

**Methods:**

We collected cervical and anal swabs, as well as oral rinse specimens from 645 sexually active inner-city young females attending a large adolescent health-clinic in New York City that offers free care and HPV vaccination. Specimens were tested for HPV-DNA using a MY09/MY11-PCR system. Type-specific prevalence of HPV at each anatomic site was compared for individuals by vaccination dose using generalized estimating equation logistic regression models.

**Results:**

The majority of subjects reported being of non-Caucasian (92%) and/or Hispanic ethnicity (61%). Median age was 18 years (range:14–20). All had practiced vaginal sex, a third (33%) practiced anal sex, and most (77%) had also engaged in oral sex. At enrollment, 21% had not received the vaccine and 51% had received three doses. Prevalent HPV infection at enrollment was detected in 54% of cervical, 42% of anal and 20% of oral specimens, with vaccine types present in 7%, 6% and 1% of specimens, respectively. Comparing prevalence for vaccine types, the detection of HPV in the cervix of vaccinated compared to unvaccinated adolescents was significantly reduced: HPV6/11 (odds ratio [OR] = 0.19, 95%CI:0.06–0.75), HPV16 (OR = 0.31, 95%CI:0.11–0.88) and HPV18 (OR = 0.14, 95%CI:0.03–0.75). For anal HPV, the risk of detecting vaccine types HPV6/11 (OR = 0.27, 95%CI:0.10–0.72) and HPV18(OR = 0.12, 95%CI:0.01–1.16) were significantly reduced for vaccinated adolescents however, the risk for HPV16 was not significantly decreased (OR = 0.63, 95%CI:0.18–2.20).

**Conclusion:**

HPV Prevalence is extremely high in inner-city female adolescents. Administration of the HPV vaccine reduced the risk for cervical HPV; however continued follow-up is required to assess the protection for HPV at all sites in young women with high exposure.

## Introduction

Cervical human papillomavirus (HPV) infection is the most common sexually transmitted viral infection (STI). Adolescents and young women have the highest rates of cervical HPV. The FDA-approved quadrivalent vaccine, GARDASIL® (Merck & Co.Inc., PA), targets HPV16 and 18 (high-risk types present in ∼65% of cervical cancers), as well as HPV6 and 11 (associated with most genital warts), and has the potential to reduce the burden of genital HPV and disease. The HPV vaccine clinical trials, however, focused almost exclusively on low-risk, primarily non-Hispanic Caucasian women with few sexual partners who were highly compliant with the vaccine schedule. When women who had a current infection (i.e., were DNA-positive) with an HPV vaccine type were assessed, the vaccine was shown to provide no benefit for preventing future infections by that type [1], [2], while a possible decrease in efficacy was observed among women who had evidence of prior infection [3], [4]. However, the effectiveness of the vaccine in highly exposed young women has not been adequately evaluated.

The association of HPV with anal and oropharyngeal papillomas and neoplasia is an important public health concern. Yet few clinical trials have reported on the effectiveness of the vaccine against anal or oral HPV [5], [6]. Post-marketing surveillance studies of cervical, anal and oral HPV in real-world settings and high-risk populations are critically important to evaluate the public health benefit of HPV vaccination to all segments of the population.

Subsequent to the approval and release of GARDASIL, we initiated a cohort study of sexually active inner-city, mostly minority, adolescent females attending the largest adolescent-specific primary care facility in the U.S. – the Mount Sinai Adolescent Health Center in New York City; that provides free health services, including HPV vaccination. The data obtained from this study will be essential to understanding the real-world impact of HPV vaccination in high-risk adolescents, and for determining future cervical cancer prevention and screening practices in cohorts of women with different HPV vaccine coverage.

## Methods

### Objective

In this report, we present data on the prevalence of HPV in this high-risk population and describe the cross-sectional association between the detection of cervical, anal and oral HPV with vaccine exposure.

### Study population and setting

The Mount Sinai Adolescent Health Center (MSAHC) uses a unique model that integrates medical, dental, sexual/reproductive, mental health and health education services. All services are confidential and free to patients. It is located on the border of East Harlem and supports an underserved population of children, adolescents, and young adults (ages 10–24); ∼80% of who are females, come from all five Boroughs of New York City: 50% from Central and East Harlem, and other parts of Manhattan, 29% from the Bronx, and 20% from other Boroughs.

### Eligibility criteria

Women are eligible to participate if they: 1) are between 12 and 19 years of age at time of consent, 2) have ever engaged in vaginal or anal intercourse, and 3) intend to get or have already received the FDA approved HPV vaccine (GARDASIL®). Women pregnant at time of recruitment or who have terminated a pregnancy within the last 4 weeks are excluded.

### Subject recruitment

Adolescent women presenting to the MSAHC are informed about the study by research staff. Study flyers are also handed out instructing women attending the adolescent-health clinic to contact research staff if they are interested in participating. In addition, MySpace® and Facebook® social-networking pages were created as health education tools to explain facts related to HPV in more detail (www.myspace.com/hpvinfo; www.facebook.com/MSAHC).

### Ethics

Written informed consent is collected from all participants prior to enrollment. This study was approved by the Institutional Review Board at Mount Sinai School of Medicine and the Committee for Clinical Investigations at Albert Einstein College of Medicine. Initially, a waiver for parental consent was approved only for women ≥18 years of age; this was later extended to adolescents aged ≥14 years, and most recently to those 12–13 years of age as per clinic policies.

### Clinical history and physical exam

All study participants receive a comprehensive gynecological examination that includes: sexual, reproductive, behavioral and psychosocial history, immunization update, blood and urine testing/screening (as indicated), and anticipatory guidance and age-specific health education. Screening for Chlamydia, gonorrhea and Syphilis is done routinely, and Herpes if symptoms are present.

### Research questionnaires

A self-administered questionnaire consisting of items to assess risk behaviors for HPV acquisition includes questions on: sexual behaviors, history of STIs and warts, characteristics of sexual partners, condom use, use of alcohol and illicit substances for participants and their sexual partners. The questionnaire is reviewed by a study coordinator at the time of the visit and any items not completed are queried. The enrollment clinical interview and questionnaire data were combined to describe the population and risk factor profiles for this analysis.

### Mucosal specimens for HPV testing

Specimen collection is performed by MSAHC clinicians. Cervical cells are collected using an endocervical Cytobrush® placed in PreservCyt transport medium (ThinPrep®, Hologic, MA) medium. Anal cells are also collected in PreservCyt using a Dacron swab moistened in tap water. Oral cell samples are collected by oral rinse and gargle using a Scope® mouthwash (Proctor&Gamble, OH). Specimens are stored at −20°C immediately following collection. Additional cervical and anal specimens are collected for Pap cytology at enrollment.

### HPV DNA genotyping

The polymerase chain reaction (PCR) detection and typing of HPV-DNA has been described in detail elsewhere [7]. Briefly, cervical, anal and oral samples are processed in a BioSafety Cabinet in a laboratory physically separated from where the PCR amplification is performed. DNA is purified from PreservCyt material by pelleting the cellular material, digestion with proteinase-K and Laureth-12, and precipitation with ethanol. Mouthwash samples are treated with SDS/proteinase-K and the DNA is phenol/chloroform extracted as described [8]. Five µl of purified DNA is then amplified by PCR using Gold-Taq with a mix of MY09/MY11 L1 consensus primers, which amplifies a 450 bp HPV-DNA fragment, and a control primer set (PC04/GH20), which simultaneously amplifies a 268 bp cellular beta-globin DNA fragment as an internal control for amplification [7], [9]. Ten µl of the PCR reaction mix is analyzed by gel electrophoresis in 3% NuSieve/0.5% SeaKem agarose (FMC BioProducts,ME), a photo taken of the ethidium stained gel, which is then transferred to nylon filters (Immobilon, Millipore,MA). The filters are hybridized overnight with radio-labeled generic probes for HPV and an oligonucleotide for β-globin as described. The membranes are washed and exposed to X-ray film (i.e., Southern blot).

Samples hybridizing to the β-globin probe but negative for the generic probe are considered HPV negative. PCR products positive by Southern blot and any sample having a specific DNA fragment migrating at ∼450 bp are analyzed for HPV-DNA type. Filters are individually hybridized using biotinylated type-specific oligonucleotide probes for multiple HPV including vaccine types HPV 6, 11, 16 and 18. Samples that test positive by the generic probe mix but negative by all type-specific probes are considered to represent “uncharacterized” HPV types. Hybridization signals of the HPV type-specific probes are recorded using a 1–5+ validated scale for signal intensity [10].

### Statistical methods

Clinical and demographic characteristics of the study cohort were examined using frequency distributions. Associations between risk factors and HPV detection, including vaccine and related HPV types, were estimated by odds ratios (OR) and 95% confidence intervals (CI) using multivariable logistic regression. In addition, to examine the vaccine effect over multiple HPV types, we fit two models using a previously described generalized estimating equation (GEE) approach [11]. The first model compared fully vaccinated subjects (three doses) to unvaccinated subjects, while the second involved all subjects with the vaccine doses modeled as an ordinal variable. In both models, the association between vaccine exposure and detection of HPV 6/11, 16, 18 and non-vaccine HPV types were modeled concurrently such that the vaccine effect varies by type, and within subject correlation was adjusted for using an exchangeable correlation structure. We mutually adjusted for all other significant risk factors identified by backward elimination, and selected confounders using a change-in-point estimate criterion for the vaccine effect [12]. Other (non-vaccine) HPV types detected were assessed individually and grouped by cancer risk potential, including other high-risk (HR) types: HPV31, 33, 35, 39, 45, 51, 52, 56, 58, and 59 reported in recent reviews [13], [14], [15]. Low-risk HPVs included other non-oncogenic types from the *alpha* PV genus and uncharacterized HPV types. Related HR-HPV for vaccine type 16 included HPV31, 33, 35, 52, and 58 (from the *alpha*-9 genus) and HPV18-related types 39, 45 and 59 (from the *alpha*-7 genus). No HPV6/11-related HPV types (from the *alpha*-10 genus) were specifically identified. Statistical analyses were conducted with the STATA 12.0 and SAS 9.2 statistical software packages.

## Results

The cohort consisted of a predominantly minority population with a median age of 18 years who had not completed high-school at the time of enrollment (**Table 1**). Only eight percent of subjects identified themselves as Caucasian. All subjects reported vaginal intercourse, some (33.2%) reported anal intercourse, and most (76.6%) also had oral-to-genital or oral-to-anal sex. The majority of subjects (68.5%) had had at least three sexual partners in their lifetime, and 41.6% had five or more partners. The overall median age at first vaginal intercourse was 15 years (range:10–19). Participants who enrolled before the age of 16, however, had a median age at first intercourse of 14 years. Over half of the subjects (54.9%) had initiated sexual intercourse a year or more before vaccination.

**Table 1:** Demographic and sexual history characteristics at enrollment (N = 645).

Cohort characteristics	N*	(%)
**Age at enrollment**		
14–15	51	(7.9%)
16–17	199	(30.9%)
18–19	395	(61.2%)
Race		
Non-Caucasian minority†	593	(91.9%)
Ethnicity		
Hispanic†	392	(60.8%)
Education		
10^th^ or lower	133	(20.6%)
11–12^th^ grade	252	(39.1%)
High-school graduate/GED	153	(23.7%)
Some college	99	(15.4%)
**Sexual activity**		
Lifetime number of male sexual partners		
1	99	(15.4%)
2	104	(16.1%)
3–4	174	(27.0%)
5–9	183	(28.4%)
10–60	85	(13.2%)
Age at first intercourse		
10–13	122	(18.9%)
14–15	307	(47.6%)
16–17	187	(29.0%)
18–19	29	(4.5%)
Number of partners in prior 3 months		
0	76	(11.8%)
1	416	(64.5%)
2	107	(16.6%)
3–8	46	(7.1%)
Anal intercourse ever		
No	431	(66.8%)
Yes	214	(33.2%)
Lifetime number of anal sex partners		
1	112	(17.4%)
2+	48	(7.4%)
Age at first anal intercourse		
13–15	33	(5.1%)
16–19	120	(18.6%)
Oral to genital sex		
Never	142	(22.0%)
Ever	490	(76.0%)
Oral to anal sex		
Never	617	(95.7%)
Ever	15	(2.3%)
Age first gave oral sex (years)		
7–15	191	(29.6%)
16–20	308	(47.8%)
Age first received oral sex (years)		
7–15	261	(40.5%)
16–19	312	(48.4%)
**Vaccine doses received**		
None (vaccine naïve)	132	(20.5%)
1 dose	97	(15.0%)
2 doses	89	(13.8%)
3 doses	327	(50.7%)
**Interval btw. 1^st^ sex intercourse and 1^st^ vaccine dose**		
4+ years prior to vaccination	81	(12.6%)
3–4 years prior to vaccination	71	(11.0%)
2–3 years prior to vaccination	82	(12.7%)
1–2 years prior to vaccination	120	(18.6%)
<1 year from date of vaccination	203	(31.5%)
>1 year post vaccination	68	(10.5%)
**History of contraceptive use**		
Withdrawal/Rhythm method	356	(55.2%)
Condom use during vaginal sex		
Never	77	(11.9%)
Rarely/Sometimes	228	(35.4%)
Most of the time	201	(31.2%)
Always	129	(20.0%)
**Ever use of prescribed contraceptives** ‡		
Oral contraceptive pill	340	(52.7%)
Vaginal ring	164	(25.4%)
Depo-Provera shot	149	(23.1%)
**History of pregnancy at entry** (N = 515)		
Ever (including non-full term)	161	(31.3%)
**History of sexually transmitted infections** ‡		
Chlamydia	232	(36.0%)
Bacterial vaginosis	174	(27.0%)
Trichomoniasis	49	(7.6%)
Gonorrhea	37	(5.7%)
Genital warts	34	(5.3%)
**Cigarette smoking**		
Never	372	(57.7%)
Yes (no longer)	177	(27.4%)
Yes (current user)	92	(14.3%)
**Marijuana smoking**		
Never	263	(40.8%)
Yes (no longer)	193	(29.9%)
Yes (current user)	178	(27.6%)
**Anal Pap** (N = 600)		
Within normal limits	547	(91.2%)
Atypical cells of undetermined significance	48	(8.0%)
Low grade squamous intraepithelial lesions	5	(0.8%)
High grade squamous intraepithelial lesions	0	(0.0%)
**Cervical Pap** (N = 611)		
Within normal limits	440	(72.0%)
Atypical cells of undetermined significance	109	(17.8%)
Low grade squamous intraepithelial lesions	61	(10.0%)
High grade squamous intraepithelial lesions	1	(0.2%)

* *Category totals may not add up to 645 due to missing data.*

† *Includes African-American, Native American, Pacific or Caribbean island, and Asian descent. Race and ethnicity are overlapping.*

‡ *Subjects may have acquired more than one STI or used more than one contraceptive method.*

History of STIs in the population was high including Chlamydia (36.0%), trichomoniasis (7.6%), gonorrhea (5.7%), and genital warts (5.3%). Almost half (47.3%) of adolescents reported using condoms ‘never’, ‘rarely’ or only ‘sometimes’ when practicing vaginal sex, and many (55.2%) also reported using the withdrawal or rhythm method for contraception. The majority of adolescents, however, used some form of recurrent prescription-based contraception including oral contraceptive pills (52.7%), vaginal rings (25.4%) or Depo-Provera injections (23.1%). More than a quarter (31.3%) of subjects reported having been pregnant.

### HPV type-specific prevalence

**Figure 1:**
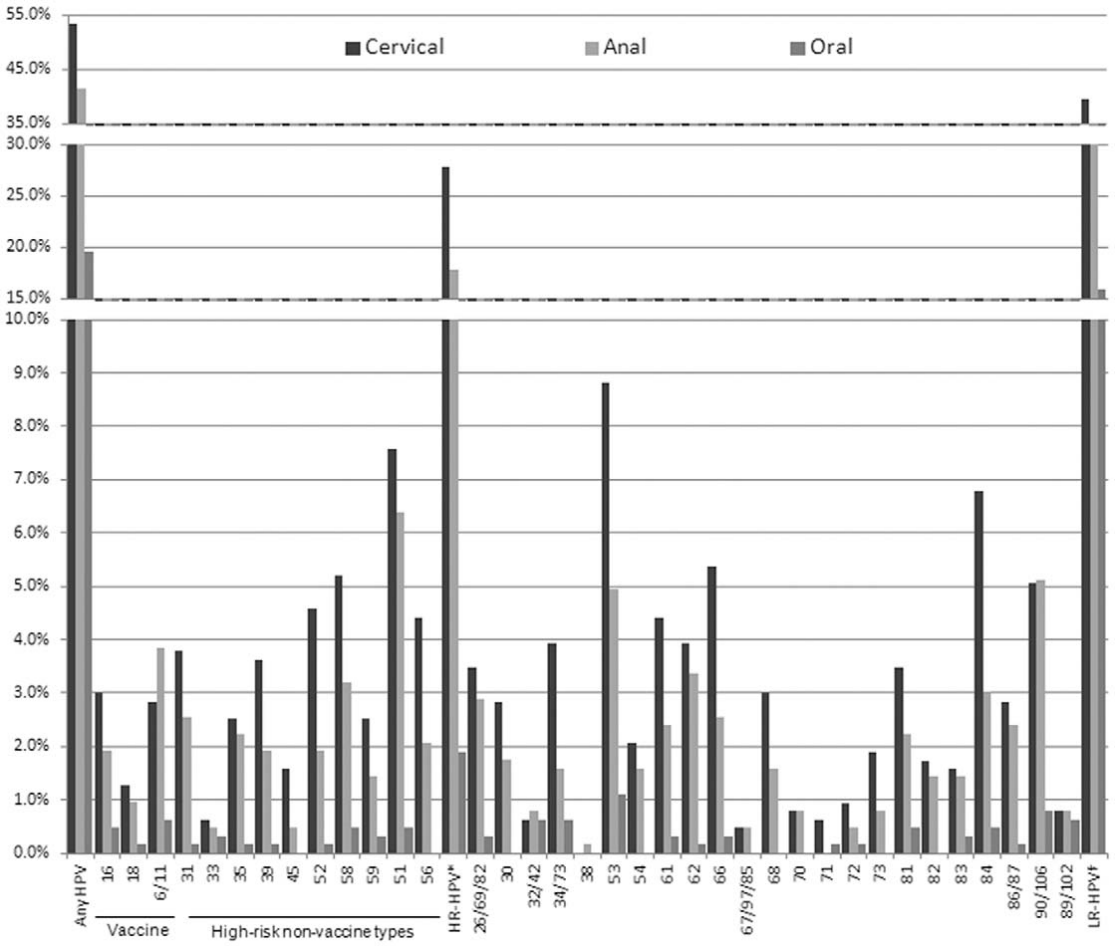
HPV type distribution at enrollment among all adolescents. Key: * Any high-risk (HR) HPV type excluding HPV16/18. † Any low-risk (LR) HPV type excluding HPV6/11.

Overall HPV-DNA detection at enrollment was 53.5% in the cervix, 41.5% in the anal canal, and 19.6% in the oral cavity. The prevalence of at least one vaccine type (i.e., HPV6/11/16/18) was 6.6%, 6.2% and 1.3% in the cervical, anal and/or oral specimens, respectively. Among the most commonly detected cervical HPV types (i.e., >5% type-specific prevalence) were HR-HPV types HPV51 and 58, as well as low-risk types 53, 66, 84 and 90/106 (**Figure 1**). The most common HPV types in the cervix were often also the most common types detected in the anal canal (including HPV51, 53, 58, 84 and 90/106, which were detected in >3% of specimens). Nevertheless, the type-specific cervical and anal concordance within individuals for these types was variable (range for non-vaccine HR-HPV types: 8.3%–42.4% and 25.0%–40.0% for vaccine types). Combined, the prevalence of HR-HPV types excluding HPV16/18 in the cervix was 27.9%, while detection levels of HR-HPV types in the anal canal and oral cavity were 17.9% and 1.9%, respectively. Co-infection with vaccine types 16 or 18 in the cervix was found in 13.9% (N = 26/187) of HR-HPV positive subjects, and corresponding co-infection rates in the anal canal and oral cavity were 18% and 25%, respectively.

### Risk factors for cervical and anal HPV detection

We examined the risk factors for HPV-vaccine, vaccine-related HR-types and all HPV types in the cervix or anal canal, using logistic regression adjusting only for vaccine dose (**Table 2**). Significant associations were observed for detection of any HPV in the cervix with increasing lifetime and recent number of male vaginal sex partners, anal intercourse, oral to anal sex, use of Depo-Provera injections and history of Chlamydia. Detection of anal HPV was also associated with higher lifetime and recent number of anal and vaginal sex partners, younger age at first anal intercourse, and history of Chlamydia and anogenital warts. No statistically significant risk associations were observed with oral HPV (not shown) due to small numbers of positive cases.

**Table 2:** Risk factors for HPV detection*.

	Cervical HPV						Anal HPV					
Characteristics	Vaccine types (6/11/16/18)		Related HR-types		All types		Vaccine types (6/11/16/18)		Related HR-types		All types	
**Age at entry**												
14–15	1.0	-	1.0	-	1.0	-	1.0	-	1.0	-	1.0	-
16–17	3.0	0.4–24.5	1.9	0.8–4.5	1.5	0.8–2.7	2.9	0.4–23.3	1.6	0.6–4.3	1.1	0.6–2.1
18–20	4.4	0.6–33.7	1.5	0.7–3.6	1.4	0.8–2.5	3.9	0.5–29.8	1.2	0.4–3.1	1.3	0.7–2.3
Ethnicity												
Non-Hispanic	1.0	-	1.0	-	1.0	-	1.0	-	1.0	-	1.0	-
Hispanic	0.8	0.4–1.4	1.0	0.6–1.4	0.8	0.6–1.1	0.7	0.3–1.3	1.0	0.6–1.7	0.8	0.5–1.0
**Sexual activity**												
Lifetime # of male vaginal sex partners												
1	1.0	-	1.0	-	1.0	-	1.0	-	1.0	-	1.0	-
2	1.4	0.4–5.3	1.4	0.6–3.0	2.1	1.2–3.7	0.7	0.1–4.0	0.6	0.2–1.9	1.0	0.5–1.8
3–4	2.0	0.6–6.8	**2.0**	**1.0–3.9**	**3.7**	**2.1–6.3**	2.4	0.7–9.1	2.1	0.9–4.9	**2.0**	**1.2–3.4**
5+	2.4	0.8–7.4	**2.1**	**1.0–3.9**	**4.0**	**2.4–6.6**	3.4	1.0–11.6	1.8	0.8–4.0	**2.1**	**1.3–3.5**
Age at first vaginal intercourse												
16+	1.0	-	1.0	-	1.0	-	1.0	-	1.0	-	1.0	-
14–15	1.0	0.5–2.1	1.2	0.8–1.8	1.0	0.7–1.4	1.7	0.8–3.9	1.4	0.8–2.6	0.9	0.6–1.2
<14	1.4	0.6–3.4	0.8	0.4–1.5	1.1	0.7–1.7	2.0	0.8–5.3	**2.1**	**1.1–4.1**	1.0	0.7–1.6
Number of partners (prior 3 months)												
0	1.0	-	1.0	-	1.0	-	1.0	-	1.0	-	1.0	-
1	0.6	0.2–1.5	0.9	0.5–1.7	1.0	0.6–1.6	1.2	0.4–3.6	0.8	0.4–1.7	1.4	0.8–2.5
2+	1.6	0.6–4.2	1.3	0.6–2.5	**2.2**	**1.2–3.9**	2.0	0.6–6.4	0.9	0.4–2.1	**1.8**	**1.0–3.3**
Anal intercourse ever												
No	1.0	-	1.0	-	1.0	-	1.0	-	1.0	-	1.0	-
Yes	1.2	0.6–2.2	1.2	0.8–1.9	**1.5**	**1.1–2.1**	**2.0**	**1.0–3.9**	**1.9**	**1.2–3.1**	**1.5**	**1.0–2.1**
Lifetime number of anal sex partners												
0	1.0	-	1.0	-	1.0	-	1.0	-	1.0	-	1.0	-
1	1.1	0.5–2.4	1.0	0.6–1.7	1.6	1.0–2.4	1.2	0.5–2.9	1.0	0.5–1.9	1.1	0.7–1.7
2+	0.6	0.1–2.9	1.2	0.6–2.4	1.1	0.6–1.9	**2.8**	**1.1–7.4**	**2.9**	**1.4–6.0**	1.6	0.9–3.0
Age at first anal intercourse												
Never	1.0	-	1.0	-	1.0	-	1.0	-	1.0	-	1.0	-
16+	1.1	0.5–2.5	1.0	0.6–1.7	1.3	0.8–1.9	1.3	0.5–2.9	1.2	0.6–2.3	1.0	0.7–1.7
<16	0.9	0.2–4.2	1.3	0.6–2.9	1.9	0.9–4.1	**3.2**	**1.1–9.2**	**4.5**	**2.0–9.9**	**2.4**	**1.2–5.1**
Oral to genital sex												
Never	1.0	-	1.0	-	1.0	-	1.0	-	1.0	-	1.0	-
Ever	1.3	0.6–3.0	1.0	0.6–1.5	1.0	0.7–1.5	1.3	0.6–3.1	1.2	0.7–2.3	1.2	0.8–1.8
Oral to anal sex												
Never	1.0	-	1.0	-	1.0	-	1.0	-	1.0	-	1.0	-
Ever	**4.2**	**1.3–13.7**	**4.6**	**1.6–13.5**	**5.4**	**1.2–24.8**	**3.6**	**1.0–13.0**	2.5	0.7–8.4	2.6	0.9–8.1
Age first gave oral sex												
Never	1.0	-	1.0	-	1.0	-	1.0	-	1.0	-	1.0	-
16+	1.9	0.7–5.1	1.3	0.7–2.3	1.4	0.9–2.2	1.8	0.6–6.0	1.2	0.6–2.4	1.3	0.8–2.0
<16	1.7	0.7–4.3	1.4	0.8–2.4	1.3	0.9–2.0	**2.9**	**1.0–8.4**	0.9	0.5–1.8	1.3	0.8–1.9
Age first received oral sex												
Never	1.0	-	1.0	-	1.0	-	1.0	-	1.0	-	1.0	-
16+	1.6	0.4–5.7	1.2	0.6–2.4	1.2	0.7–2.0	1.6	0.4–5.5	1.2	0.5–2.7	1.3	0.7–2.3
<16	1.3	0.4–4.7	1.3	0.7–2.6	1.3	0.8–2.1	1.3	0.4–4.7	1.0	0.4–2.2	1.4	0.8–2.5
**Vaccine doses received** †												
None (vaccine naïve)	1.0	-	1.0	-	1.0	-	1.0	-	1.0	-	1.0	-
3 doses	**0.2**	**0.1–0.4**	0.8	0.5–1.3	0.7	0.4–1.0	**0.3**	**0.2–0.7**	0.7	0.4–1.3	0.7	0.5–1.0
**Interval btw 1^st^ sex and vaccination**												
2+ years prior to vaccination	2.4	0.5–11.3	1.2	0.6–2.7	**1.9**	**1.0–3.4**	6.8	0.9–54.4	1.7	0.5–5.3	1.1	0.6–2.0
1–2 years prior to vaccination	0.6	0.1–3.7	1.2	0.5–2.7	1.4	0.8–2.6	2.2	0.2–20.9	1.7	0.5–5.7	0.9	0.5–1.6
Within 1 year of vaccination	1.1	0.2–5.5	1.4	0.7–3.0	1.4	0.8–2.5	2.0	0.2–17.2	2.6	0.9–7.7	1.1	0.6–2.0
>1 year post vaccination	1.0	-	1.0	-	1.0	-	1.0	-	1.0	-	1.0	-
**Prescribed contraceptives**												
Depo-Provera shot never	1.0	-	1.0	-	1.0	-	1.0	-	1.0	-	1.0	-
Depo-Provera shot ever	1.5	0.7–3.1	**1.5**	**1.0–2.4**	**1.5**	**1.0–2.2**	1.6	0.8–3.4	**1.9**	**1.1–3.2**	1.2	0.8–1.8
**History of STI**												
Chlamydia never	1.0	-	1.0	-	1.0	-	1.0	-	1.0	-	1.0	-
Chlamydia ever	**2.5**	**1.3–4.9**	1.1	0.7–1.7	**2.2**	**1.6–3.1**	**2.2**	**1.1–4.3**	**1.7**	**1.1–2.8**	**2.2**	**1.6–3.1**
Genital warts never	1.0	-	1.0	-	1.0	-	1.0	-	1.0	-	1.0	-
Genital warts ever	**2.9**	**1.1–7.7**	1.2	0.5–2.7	1.9	0.9–3.9	**5.4**	**2.2–13.1**	**1.0**	**0.3–3.0**	**3.0**	**1.4–6.4**

* *Odds ratio (OR) and 95% confidence intervals (CI) shown were derived by logistic regression adjusting for vaccine status only.*

† *Due to small numbers, estimates are only shown for the comparison between fully vaccinated to unvaccinated subjects, excluding those entering study with 1 or 2 doses.*

**Table 3:** Adjusted associations with vaccine exposure for detecting a type-specific HPV infection.

HPV types	Cervical HPV		Anal HPV	
	OR 3 doses (95%CI)*	p-value*	OR 3 doses (95%CI)*	p-value*
HPV6/11	0.19 (0.06–0.59)	0.004	0.27 (0.10–0.72)	0.009
Other low-risk types excluding HPV6/11	0.56 (0.36–0.85)	0.007	0.84 (0.52–1.35)	0.470
HPV16	0.31 (0.11–0.88)	0.028	0.63 (0.18–2.20)	0.469
HPV18	0.14 (0.03–0.75)	0.022	0.12 (0.01–1.16)	0.067
Other high-risk types excluding HPV16/18	0.76 (0.47–1.22)	0.254	0.65 (0.39–1.11)	0.115

* *Odds ratios (OR), 95% confidence intervals (CI) and p-values are shown for vaccine effect on HPV detection for each HPV type (or types) comparing fully vaccinated to unvaccinated subjects excluding those presenting with 1 or 2 doses at enrollment. The estimates were derived by multivariate generalized estimating equation (GEE) with logistic regression mutually adjusting for all concurrent types, age, ethnicity, number of vaginal sex partners, and history of chlamydia.*

Except for vaccine exposure, similar risk factor associations were observed for detection of vaccine types alone and related HR-HPV (*alpha*-9 and -7) types. Whereas vaccine types were significantly less likely to be detected in the cervix and anal canal with any vaccine exposure, this was not observed for other related HR-HPV types or all types combined. Subjects who had engaged in vaginal sex more than two years prior to vaccination were also more likely to present with an HPV infection independent of vaccine dose.

### Impact of vaccination on detection of cervical, anal and oral HPV

**Figure 2:**
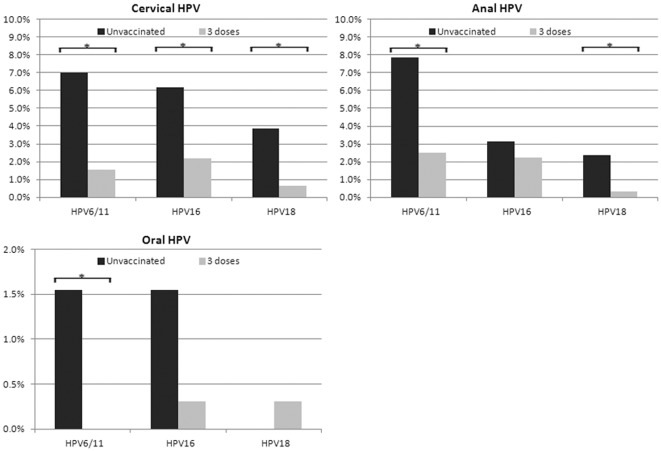
Site specific prevalence of HPV vaccine types by vaccine exposure. Key: HPV types showing significant differences (by Fisher's exact test p<0.1) are indicated by starred brackets.

At enrollment, 20.5% of subjects had yet to receive the HPV vaccine, 15.0% had received their first dose, 13.8% a second dose, and 50.7% all three doses. Comparing prevalence for HPV vaccine types among adolescents entering the study with all three vaccine doses compared to unvaccinated subjects, we observed significantly lower HPV6/11 detection for fully vaccinated compared to unvaccinated subjects in the cervix (OR = 0.21, Fisher's exact test p = 0.005), anal canal (OR = 0.30, p = 0.013), and oral cavity (OR≈0.08, p = 0.081; **Figure 2**). Gradual declines in HPV6/11 prevalence were also found with increasing dose from none to three doses for the cervix (Wilcoxon rank sum test p for trend = 0.004) and anal canal (p-trend = 0.009). With respect to HR-HPV vaccine types 16 and 18, we observed a significantly lower prevalence with vaccination in the cervix (OR = 0.34, p = 0.042, p-trend = 0.074; and OR = 0.16, p = 0.023, p-trend = 0.022, respectively). In contrast, whereas the decline in anal HPV18 was significant (OR = 0.13, p = 0.073, p-trend = 0.033), the decrease in anal HPV16 was not (OR = 0.69, p = 0.519, p-trend = 0.924). Detection of HPV16 and 18 in the oral cavity was low overall and non-existent among subjects with one or two vaccine doses. Nonetheless, these cross-sectional data raise the possibility that HPV vaccination might have less impact on extra-cervical HR-HPV types when administered to a sexually active high-risk population. These associations, however, were not adjusted for sexual activity or other concurrent HPV type infections. To address this, we performed a GEE multivariable logistic regression analysis.

**Table 3** shows the relative risk associations estimated by OR for HPV detection by vaccine exposure mutually adjusting for concurrent HPV types, as well as other epidemiological risk factors and confounders. We observed statistically significant risk reductions in detecting HPV types 16, 6/11 and 18 in the cervix of 69%, 81% and 86%, respectively, among adolescents who had three doses compared to those who had no vaccination. However, a statistically non-significant 37% risk reduction for detecting HPV16 in the anal canal was observed amongst fully vaccinated adolescents compared to non-vaccinated adolescents (OR = 0.63, 95%CI:0.18–2.20), whereas significant reductions of 73% and 88% were seen for HPV6/11 and HPV18, respectively. When vaccine exposure was modeled by dose (including subjects with only one or two doses at enrollment), we found that the odds of detecting HPV6/11 per vaccine dose were significantly lower for the cervix (OR_dose_ = 0.54, 95%CI:-.36–0.83) and anal canal (OR_dose_ = 0.61, 95%CI:0.43–0.86), as well for HPV18 in the anal canal (OR_dose_ = 0.46, 95%CI:0.23–0.93).

**Figure 3:**
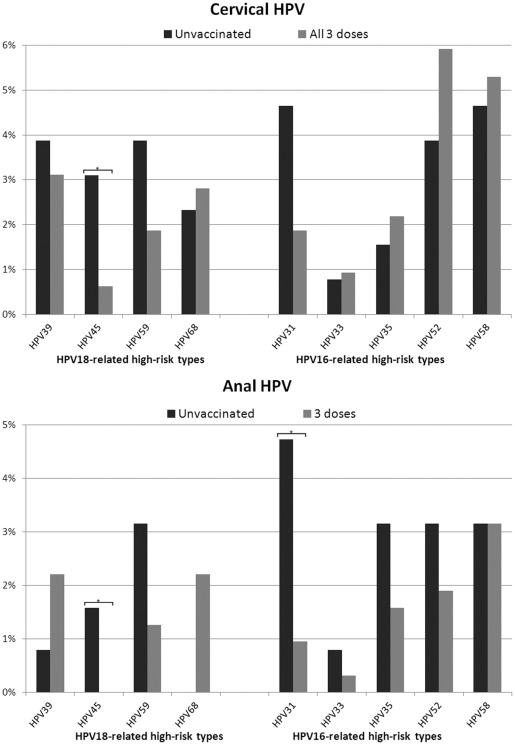
Site specific prevalence of HPV vaccine-related types by vaccine exposure. Key: HPV types showing significant differences (by Fisher's exact test p<0.1) are indicated by starred brackets.

Independent of HPV16 and 18, the adjusted OR for detecting other HR-HPV types combined did not show significance. We further assessed the type-specific prevalence for vaccine related HR-HPV types from within the *alpha*-9 and -7 genera by vaccine exposure (**Figure 3**). Significant declines in detection were observed for HPV18-related type 45 (OR = 0.20, 95%CI:0.0–0.9, p-trend = 0.018) and HPV16-related type 31 (OR = 0.39, 95%CI:0.1–1.2, p-trend = 0.027) in the cervix, and in the anal canal (OR = 0.0, 95%CI:0.0–0.8, p-trend = 0.027; and OR = 0.19, 95%CI:0.1–0.7, p-trend = 0.009, respectively). Similar declines, albeit not significant, were observed at both sites only for HPV59. Furthermore, although not significant, an increased prevalence of cervical HPV35, 52 and 58 was seen in vaccinated individuals; similar increases were recently reported for vaccinated individuals in two studies [16], [17].

## Discussion

The HPV quadrivalent vaccine has been shown to have high efficacy against infection as well as HPV16/18-positive cervical pre-cancer and HPV6/11-positive genital warts [2], [18]. However, the vaccine trials focused exclusively on low risk, mostly non-Hispanic Caucasian women with few (no more than four) sexual partners, who had no history of abnormal cervical cytology, cervical disease or genital warts, and who were highly compliant with the vaccine schedule [2], [18], [19], [20]. In this article, we assessed the HPV burden in a high-risk urban minority adolescent female population, and describe the cross-sectional association between the detection of cervical and extra-cervical HPV with vaccine exposure. The cross-sectional data shown here indicates that there is continued detection of cervical HPV with vaccine types after vaccination. This raises questions regarding the ‘real-world’ impact of vaccinating high-risk populations (unlike those studied in the vaccine trials) with potentially frequent prior exposures to vaccine types as well as to other HR-HPV types. Moreover, studying a high-risk population of sexually active adolescent women, we also observe a high prevalence of extra-cervical HPV among both vaccinated and unvaccinated individuals. These observations have important public health implications for future screening and prevention of HPV-related cancer in a high-risk population.

The reported study has strengths and limitations. The current study evaluated HPV at multiple anatomical sites associated with sexual exposure. While it is generally understood that acquisition and prevalence of cervical HPV is at its highest in adolescent women, how this relates to the prevalence and acquisition of HPV in other tissues susceptible to HR-HPV associated tumorigenesis (i.e., the anal epithelium and oral cavity), is not currently known. Among the limitations, it should be noted that because we are specifically targeting sexually active adolescent females, either at the time they receive the HPV vaccine or soon thereafter, our population is older than the targeted age for vaccination, but covers the age range recommended for catch-up vaccination (www.cdc.gov/std/hpv/STDFact-HPV-vaccine-hcp.htm). In addition, we assessed cross-sectional associations between HPV-DNA infection, vaccine exposure and risk factors. As such, the history of HPV exposure prior to vaccination is not known for those individuals enrolled after vaccine initiation. Furthermore, it is impossible to ascertain via HPV-DNA detection alone if test positivity is equated with true (active, albeit latent) viral infection that may cause neoplastic disease. With longitudinal follow-up, we will be able to assess the HPV incidence and risk factors for *“breakthrough”* persistent infections (i.e., repeated detection of an HPV vaccine type in someone who was previously HPV-DNA negative for that type at enrollment) [21].

When compared to the general MSAHC clinic population (data not shown), the study cohort is younger (mean patient age in 2010 was 18.6 vs. 17.7 in this study) but comparable in terms of prior STIs. The high-risk nature of the population is also evident in the observed prevalent HPV detection. Among the strongest risk factors for detection of cervical and anal HPV (independent of vaccination) were number of sexual partners, anal intercourse, oral-to-anal sex, receipt of a Depo-Provera injection, and history of Chlamydia. Vaccination was significantly associated with cervical and anal HPV detection only after the vaccine types were assessed separately.

Recent evidence suggests the HPV vaccines will impact both cervical and anal HPV co-incidence rates, although efficacy against anal HPV depends on cervical HPV positivity [5], [6], [22]. Interestingly, whereas detection of anal HPV vaccine types 6/11 and 18 were significantly lower among vaccinated individuals in this study, the corresponding decrease for HPV16 was not. This was independent of the presence of other HPV types, prior sexual activity and other risk factors. A lower efficacy was observed against persistent anal infection by HPV16 (54.0%) compared to HPV18 (73.6%) in the quadrivalent HPV vaccine male trial intent-to-treat analyses [5]. Lower efficacy rates have also been reported in women against anal HPV16 and 18 infections (68.2% and 55.5%, respectively) compared to the cervix (75.8% and 78.6%, respectively) for the bivalent HPV vaccine [23]. HPV16 in particular has been shown to be associated with the majority of anal and oropharyngeal neoplasias. The implications of HPV infection at non-cervical sites on vaccine efficacy, however, remain to be evaluated. Moreover, while testing for HPV is approved as an adjunct screening test to Pap cytology for the cervix, testing of other sites (anal or oral) is not routinely performed.

Finally, whereas the odds of detecting HPV vaccine types in the cervix decreased significantly among vaccinated adolescents, the odds of detecting other vaccine related and un-related HR-HPV types did not show consistent decreases with vaccination. We observed significant univariate declines in detection only for HPV16 and 18-related HR-HPV types 31 and 45 in the cervix and anal canal, respectively, as did other studies. While history of previous infection with a vaccine type could not be controlled for in the analyses, it is unlikely the result is solely due to prior exposure given the observed prevalence among unvaccinated individuals. Vaccine efficacy has been shown against persistent infection in the trial cohorts for HPV33, 31, 45 and 51 (with or without HPV16/18 co-infection) [16], [17]. The detection of other common HR-HPV types in vaccinated populations that will remain at a high risk of disease has implications for future preventative and screening strategies.

In summary, our data to date suggest continued detection of cervical and extra-cervical infection with HPV vaccine types after vaccination, in addition to other HR-HPV types. This study provides the ‘real-world’ impact of vaccinating high-risk adolescent populations (unlike those studied in the vaccine trials) with potentially frequent prior exposures to HPV vaccine types, as well as to related HR-HPV types. Findings from studies such as this are therefore critical to document the continued burden of HPV and to properly design future multi-type prophylactic HPV vaccines and continued screening strategies to prevent HPV-related disease.
